# First results with SiPM tiles for TOF PET based on FBK RGB-HD technology

**DOI:** 10.1186/2197-7364-2-S1-A86

**Published:** 2015-05-18

**Authors:** Alessandro Ferri, Fabio Acerbi, Peter Fischer, Alberto Gola, Giovanni Paternoster, Claudio Piemonte, Michael Ritzert, Torsten Solf, Illaria Sacco, Nicola Zorzi

**Affiliations:** Fondazione Bruno Kessler, Trento, Italy; Heidelberg University, Germany; Philips Healthcare, Germany

We present the first results of timing and energy resolution of two newly developed tiles based on FBK RGB-HD SiPMs. The first tile has dimensions of 32x32 mm^2^ and is composed of 8x8 SiPMs, with a regular pitch of 4 mm and a cell size of 25x25 μm^2^. Although manufactured with a standard bond wire technology, the tile achieves a fill factor at the tile level of 85%. We produced two versions: one with a single-ended and the other with differential readout. We tested the first prototypes with single-ended readout with a scintillator array, perfectly matching the tile pitch and composed of 8x8 LYSO crystals with dimensions of 4x4x22 mm^3^. First, we tested the tile using a single-channel setup, based on a fast, discrete amplifier, a digital oscilloscope and a PC, reading one SiPM at a time. At 20 °C, we measured an energy resolution of 10.7% FWHM. For the timing measurements we compared two conditions: when only one SiPM was biased and read, and when all the 64 SiPMs were biased but only one was read. At 20 °C, we measured a timing resolution of 200 ps FWHM in the first case, and 220 ps FWHM in the second case. Then, we tested the whole tile with a dedicated ASIC (PETA3), and measured the energy and timing resolution of two tiles in coincidence. The second tile is composed of 144 SiPMs, mounted on a water cooled, ceramic LTCC substrate. On the top side, it contains 12x12 SiPMs with a regular pitch of 2.5 mm. Also in this case, the SiPM technology is the RGB-HD with a cell size of 25x25 μm^2^. On the bottom side, four readout ASICs of the latest generation (PETA5) are flip-chip mounted. First results will be presented.

